# FGF23 levels as a marker of physical performance and falls in community-dwelling very old individuals

**DOI:** 10.20945/2359-3997000000488

**Published:** 2022-05-25

**Authors:** Mariana Zuccolotto Foroni, Maysa Seabra Cendoroglo, Aline Granja Costa, Rosangela Villa Marin-Mio, Patricia Ferreira do Prado Moreira, Sergio Setsuo Maeda, John P. Bilezikian, Marise Lazaretti-Castro

**Affiliations:** 1 Universidade Federal de São Paulo Escola Paulista de Medicina Divisão de Endocrinologia São Paulo SP Brasil Divisão de Endocrinologia, Escola Paulista de Medicina – Universidade Federal de São Paulo (Unifesp), São Paulo, SP, Brasil; 2 Universidade Federal de São Paulo Escola Paulista de Medicina Divisão de Geriatria São Paulo SP Brasil Divisão de Geriatria, Escola Paulista de Medicina – Universidade Federal de São Paulo (Unifesp), São Paulo, SP, Brasil; 3 Columbia University College of Physicians and Surgeons Department of Medicine, Division of Endocrinology New York New York USA Department of Medicine, Division of Endocrinology, College of Physicians and Surgeons, Columbia University, New York, New York, USA

**Keywords:** Fibroblast growth factor 23, physical performance, falls, muscle strength, body balance, aging, very old

## Abstract

**Objective:**

The fibroblast growth factor 23 (FGF23) has been related to biological aging, but data in elderly individuals are scant. We determined the profile of serum FGF23 levels in a population of very-old individuals and studied their correlations with parameters of bone metabolism and health markers, as functional performance.

**Materials and methods:**

This cross-sectional study was performed on 182 community dwellers aged ≥ 80 years. Serum levels of FGF23, PTH, calcium, albumin, phosphorus, creatinine, bone markers, and bone mineral density data were analyzed.  Physical performance was evaluated with the stationary march (Step), Flamingo, and functional reach tests, along with questionnaires to assess falls and fractures in the previous year, energy expenditure (MET), and the Charlson index (CI). Physical activity was evaluated with the International Physical Activity Questionnaire (IPAQ).

**Results:**

Most participants (75%) had FGF23 levels between 30-120 RU/mL (range: 6.0-3,170.0 RU/mL). FGF23 levels correlated with estimated glomerular filtration rate (eGFR; r = -0.335; p = 0.001) and PTH (r = 0.318; p < 0.0001). Individuals with FGF23 in the highest tertile had more falls in the previous year (p = 0.032), worse performance in the Flamingo (p = 0.009) and Step (p < 0.001) tests, worse CI (p = 0.009) and a trend toward sedentary lifestyle (p = 0.056). On multiple regression, FGF23 tertiles remained significant, independently of eGFR, for falls in the previous year, performance in the Flamingo and stationary march tests, lean mass index, and IPAQ classification.

**Conclusion:**

In a population of very elderly individuals, FGF23 levels were inversely associated with neuromuscular and functional performances. Higher concentrations were related to more falls, lower muscle strength and aerobic capacity, and poorer balance, regardless of renal function, suggesting a potentially deleterious role of high FGF23 concentrations in musculoskeletal health.

## INTRODUCTION

Individuals over the age of 80 represent the segment of the population with the highest relative growth (3.8% per year). By 2015, the world’s population of individuals over the age of 80 was estimated at 125 million and, according to projections, will be three times higher by 2050 (1). With this profound change in population demographics, chronic-degenerative diseases, functional dependence with a consequent reduction in quality of life, and increased social costs are presenting a challenge to public health systems worldwide. This has led, in turn, to increasing interest in the mechanisms involved in the aging process.

Some studies have found associations between proteins regulating the metabolism of phosphorus and aging (2). FGF23 is a major regulator of phosphorus metabolism by reducing the number of sodium-phosphate type 2a (NaPi-2a) cotransporters in the proximal tubules (3,4). Besides, FGF23 strongly suppresses the activity of 1-alpha hydroxylase in the kidney, decreasing the production of the active form of vitamin D [1,25(OH)_2_D] (3,4).

To bind to its specific receptor (FGFR) located on the cell membrane, FGF23 requires the presence of *klotho* protein (coreceptor) (2). In humans, *KLOTHO* gene polymorphisms are associated with biological markers of aging, such as life expectancy, cognition, bone mineral density (BMD), and cardiovascular diseases (3).

Since FGF23 receptors and/or co-receptors have been identified in extra-renal tissues like the parathyroid glands, cardiac and skeletal muscles (4,5), other actions of the FGF23/*klotho* pathway besides renal phosphorus regulation have been studied. However, data in humans pertinent to FGF23’s non-classical effects are scarce, and virtually non-existent in the aged population.

With regard to the need to elucidate the potential roles of FGF23 as a marker of aging, we measured serum levels of FGF23 in a free-living aged population of 80 years or more. We analyzed correlations between FGF23 levels and other parameters of bone and mineral metabolism as well as health markers, such as functional performance (muscle performance and balance) and the presence of comorbidities.

## SUBJECTS AND METHODS

### Study Population

This cross-sectional study was conducted in a cohort of individuals aged 80 years or older who were participating in a Geriatrics Program at Unifesp. Inclusion criteria were age ≥ 80 years, free-living, independence in performing basic activities of daily life, ability to walk without human support, and chronic health conditions under control. Patients who had moderate or severe dementia, terminal illness, hospitalization in the last 6 months, were on dialysis or refused to participate in the study were excluded. The study included 182 individuals.

### Clinical evaluation

Clinical evaluation was performed at the first visit, through a questionnaire that included personal history, identification of diseases, description and quantification of medications, calcium and vitamin D supplements taken, and life habits. The Charlson comorbidity index of each individual was calculated based on these data (6). Questions quantifying the number of falls and fractures in the previous year were answered by the participants and/or their companions. Fall was defined as an unintentional fall from a standing height. Falls due to a violent blow, loss of consciousness, a central nervous system event (e.g. stroke or seizure) were excluded. Previously reported fractures were considered only when the story was reliable. The level of physical activity was determined by the International Physical Activity Questionnaire (IPAQ, short form) (7) from which metabolic equivalent units (MET) were calculated and energy expenditure was quantified (8).

### Anthropometry

Body mass index (BMI) was calculated from measurements of weight and height and classified according to the Lipschitz criteria, as recommended for elderly individuals (9).

### Laboratory tests

Blood samples were collected from all participants in the morning after a 12-hour fast. Levels of (C-terminal) FGF23 were measured by ELISA (Immutopics Inc., San Clemente, CA, USA, Lot # 3021103; intraassay coefficient 1.4 to 2.4% and interassay coefficient 2.4 to 4.7%, as establishedby the manufacturer). The concentrations of albumin, total calcium, and phosphorus were measured by an automated colorimetric method, and those of creatinine by alkaline picrate. Concentrations of 25(OH)D were quantified by chemiluminescence (LIAISON 25 OH Vitamin D Total, DiaSorin, Stillwater, MN, USA; intraassay and interassay variation coefficients 1.6% and 5.6%, respectively). PTH was measured by immunochemiluminescence (Elecsys^®^ 2010, Roche Diagnostics, Indianapolis, IN, USA; intraassay and interassay coefficients of variation 3.0% and 3.5%, respectively) and procollagen type 1 N-propeptide (P1NP) and serum C-terminal telopeptide of type 1 collagen (CTX) by electrochemiluminescence (Elecsys^®^ 2010; intraassay and interassay coefficients of variation 1.8% and 2.7%, respectively, for P1NP and 4.6% and 4.7%, respectively, for CTX). Calcium was corrected for albumin levels (10). The estimated glomerular filtration rate (eGFR) was calculated by the CKD-EPI equation (11).

### Densitometric evaluation

Bone mineral density (BMD) and body composition were measured by Dual Energy X-ray Absorptiometry (DXA) Hologic^®^ (Discovery A, Waltham, MA, USA) performed on the lumbar spine (L1-L4), femoral neck, total hip, and total body, and the results obtained were classified according to the World Health Organization (WHO) criteria (12). The coefficients of variation were 0.8% for lumbar spine and total hip and 1.2% for femoral neck. Body composition measurements were obtained from total body densitometry. We calculated the participants’ lean mass index (total lean mass in kilograms divided by height in square meters) (13), Baumgartner index [appendicular lean mass (ALM) divided by the square height] (14) and the ALM/BMI index (appendicular lean mass in kilograms divided by body mass index) (15). Based on these last two indices, the participants were classified as having either low or adequate ALM.

### Physical tests

The following physical tests were used to predict the risk of falls:

1. **Flamingo balance test** (assessment of static balance): the individual raises one of the lower limbs forming a 90^o^ angle with the femur, with hands on the waist, and tries to maintain position for 30 seconds. The test is performed 3 times and the longest time the patient is able to maintain the position is recorded as the final score (16).
2. **Functional reach** (assessment of dynamic balance): the patient stands facing a wall with a tape measure attached to it, parallel to the floor, at the height of their acromion, with arms extended making a 90º angle to the trunk. The individual leans as far forward as possible and hold this position for 3 seconds, without lifting the heels off the floor. The distance reached is recorded and the best of 3 attempts is considered to be the final score (17).
3. **Stationary march – Step** (assessment of aerobic capacity and lower limb strength): after prior training of a simulated gait, the number of times the right knee of the individual rises to at least the midpoint of the distance between the patella and the iliac crest is counted for a total duration of 2 minutes (16,18).

This research project was approved by the Research Ethics Committee at UNIFESP (CAAE nº: 80688015.5.0000.5505) and conducted according to the principles of the Declaration of Helsinki. Informed consent was obtained from all individual participants prior to their inclusion in the study.

### Statistical analysis

FGF23 levels were analyzed as continuous variables and tertiles. Categorical variables are presented as absolute and relative frequencies, and continuous variables as measures of central tendency and dispersion (mean or median, standard deviation, tertiles, and minimum and maximum values).

The Kolmogorov-Smirnov test was used to evaluate the normality of the distribution of the variables and the chi-square test was used to evaluate the distribution of the participants by IPAQ, Baumgartner, and ALM/BMI classifications. Linear associations between two continuous variables were evaluated with Pearson’s correlation and nonparametric variables with Spearman’s correlation. Student’s *t*- test for independent samples and ANOVA were used to compare mean values between two groups and more than two groups, respectively. When differences in mean values were observed in ANOVA, such differences were identified using Duncan’s multiple comparisons. In case of violation of data normality, the mean ranks were compared using the nonparametric Mann-Whitney and Kruskal-Wallis tests alternatively. Once differences in the Kruskal-Wallis test were detected, they were identified using Dunn-Bonferroni tests.

Multiple regression models were used to evaluate the simultaneous effects of FGF23, sex, age, 25(OH)D, number of medications, number of comorbidities, and several other variables on each of the dependent variables. For the dependent variables of continuous numerical nature, linear regression models were used for continuous numerical values, and the Poisson models were used for categorical numerical values. For dependent, dichotomous variables (Baumgartner and categorical ALM/BMI), logistic regression models were used, and for polytomous values (IPAQ categories), a multinomial regression model was applied. A 5% significance level was adopted for all statistical tests. Statistical analyses were performed using SPSS 20.0 and Stata 12.

## RESULTS

The mean age of the 182 participants was 86.5 ± 4.5 years (range 80 to 99 years); 138 (75.8%) were women (86.5 ± 4.0 years) and 44 (24.2%) were men (86.5 ± 9.0 years). Table 1 describes the demographic, clinical and densitometric characteristics of the participants: most were Caucasians, overweight, 80 to 85 years old and active according to IPAQ. Only three participants (1.6%) were current smokers. All body composition parameters obtained from DXA were different between men and women (p < 0.0001). The mean ± SD %fat mass was 34.8 ± 6.9 for the total sample (men and women). The mean lean mass index was 16.34 ± 1.96 kg/m^2^ for women and 18.03 ± 1.80 kg/m^2^ for men, and the mean Baumgartner index was 6.59 ± 0.97 kg/m^2^ for women and 7.49 ± 0.91 kg/m^2^ for men. The mean ALM/BMI index was 0.551 ± 0.077 for women and 0.775 ± 0.104 for men. According to those two last indexes, most individuals (82.4% by the Baumgartner index and 57.4% by the ALM/BMI index) had an adequate ALM.

**Table 1:** Demographic, clinical and densitometric characteristics of the very old individuals participating in the study

Parameters		Mean ± SD	n	%
Sex			**182**	**100.0**
	Female		138	75.8
	Male		44	24.2
Ethnicity			**182**	**100.0**
	Caucasian		124	68.2
	African		11	6.0
	Asian		15	8.3
	Hybrid (Black/White)		31	17.0
	Indian		1	0.5
Age (years)		86.5 ± 4.5	**182**	
Age group (years)			**182**	**100.0**
	80-85		90	49.5
	86-90		56	30.8
	91-95		30	16.4
	≥96		6	3.3
BMI (kg/m^2^)		26.8 ± 4.4	**182**	
BMI – Classification			**182**	**100.0**
	Low weight (<22 kg/m²)		26	14.2
	Normal weight (22 to 26.9 kg/m^2^)		70	38.5
	Excessive weight (≥27 kg/m^2^)		86	47.3
Bone densitometry			**152**	**100.0**
	Normal		16	10.5
	Osteopenia		77	50.7
	Osteoporosis		59	38.8
Baumgartner – Classification			**148**	**100.0**
	Low lean mass (F ≤ 5.45 kg/m^2^; M ≤ 7.26 kg/m^2^)		26	17.6
	Adequate (F > 5.45 kg/m^2^; M > 7.26 kg/m^2^)		122	82.4
ALM/BMI – Classification			**148**	**100.0**
	Low lean mass (F < 0.512; M < 0.789)		63	42.6
	Adequate (F ≥ 0.512; M ≥ 0.789)		85	57.4
IPAQ – Categories			**140**	**100.0**
	Sedentary		18	12.9
	Irregularly active A		16	11.4
	Irregularly active B		20	14.3
	Active		85	60.7
	Very active		1	0.7
Number of medications		6.1 ± 2.8	**180**	
Number of comorbidities		4.4 ± 2.0	**182**	
Charlson Index		1.9 ± 1.4	**182**	
MET (kcal)		871.3 ± 680.8	**140**	
Functional Tests				
	Flamingo (s)	7.1 ± 8.4	**151**	
	Functional reach (cm)	23.7 ± 7.0	**150**	
	Stationary march (rep/2 min.)	49.7 ± 17.9	**150**	

Abbreviations: SD – standard deviation; BMI – body mass index; F – female sex; M – male sex; ALM – appendicular lean mass; IPAQ – International Physical Activity Questionnaire; MET – energy expenditure.

Table 2 presents the laboratory profile of the participants. Among the very old subjects, 94.6% had eGFR above 30 mL/min. Inadequate concentrations of vitamin D [25(OH)D < 30 ng/mL] were observed in 86.8% of the entire sample, and vitamin D deficiency (<20 ng/mL) was observed in 57.7%, whereas severe vitamin D deficiency (<10 ng/mL) was found in 13.7%. Of the total, 42.8% of the individuals were taking calcium supplementation, with doses ranging from 500 to 1,250 mg/day, and 38.5% were taking vitamin D supplementation, with doses ranging from 400 to 1,400 IU/day. Serum PTH levels were elevated (>65 pg/mL) in 35.7% of the subjects, of whom 70.8% had 25(OH)D<20 ng/mL. Three of these individuals had concomitant hypercalcemia, interpreted as having primary hyperparathyroidism (1.6%).

**Table 2:** Laboratory characteristics of the very old individuals participating in the study

Parameters	n	Mean ± SD or Median (Minimum-Maximum)	Reference value
FGF23*	182	68.0 (6.0-3170.8)	<100 RU/mL
Albumin	182	4.2 ± 0.3	3.4 to 4.8 g/dL
Corrected calcium	179	9.2 ± 0.5	8.8 to 10.6 mg/dL
Phosphorus	134	3.4 ± 0.4	F: 2.3 to 4.3 mg/dL
	44	3.0 ± 0.4	M: 2.4 to 4.6 mg/dL
25(OH)D	182	19.8 ± 9.5	>30 ng/mL
eGFR CKD	182	57.0 ± 16.1	>90 mL/min
PTH*	182	54.6 (12.9-718.4)	15 to 65 pg/mL
CTX	138	0.318 ± 0.220	F: <0.650 ng/mL
	44	0.281 ± 0.160	M: <0.850 ng/mL
P1NP	138	43.5 ± 30.0	F (postmenopausal): 16,3 a 73,9 ng/mL
	44	39.0 ± 23.5	M: 13.9 to 85.5 ng/mL

Abbreviations: SD – standard deviation; FGF23 – fibroblast growth factor 23; 25(OH)D – 25 hydroxyvitamin D; eGFR CKD – estimated glomerular filtration rate by the CKD-EPI formula; PTH –parathyroid hormone; CTX – C-terminal telopeptide of type 1 collagen; P1NP – procollagen type 1 N-propeptide.

* Nonparametric data.

An asymmetric distribution of FGF23 concentrations was verified, with 75% of the participants presenting with FGF23 values between 30 and 120 RU/mL. Concentrations above 200 RU/mL were found in 22 outliers (12%), all of whom had eGFR greater than 32 mL/min (mean 49.7 mL/min, range 32.0 to 79.0 mL/min), normal phosphorus (mean 3.3 mg/dL, range 2.4 to 4.1 mg/dL), were not current smokers, and did not take calcitriol. Hyperparathyroidism (PTH > 65 pg/mL) was observed in 11 (50%) of these individuals, including all 3 with primary hyperparathyroidism. Vitamin D deficiency (<20 ng/mL) was present in 63.6% (n = 14) of them, similar to the entire sample (57.7%).

Information on falls and previous fractures were provided by 152 individuals, of whom 76 (50%) reported 160 falls (range of 1 to 10 per individual) in the previous year. Eight fractures occurred after falling from a standing height in 6 individuals (2 men and 4 women); 2 occurred in the femur, 2 in the wrist, 1 in the lumbar vertebra, 1 in the shoulder, 1 in the rib, and 1 in the phalanx of the hand.

There was no significant difference in mean FGF23 levels between the groups according to gender, age, smoking, use of any medication related to calcium homeostasis, or densitometric diagnosis. FGF23 levels correlated significantly with several variables, like the stationary march test scores, eGFR, and serum PTH levels, as described in Table 3.

**Table 3:** Statistically significant (p < 0.05) correlations* observed between FGF23 concentrations and clinical and laboratory variables

	r	p
**Positive correlations**		
PTH	0.318	0.000
P1NP	0.294	0.000
% fat mass	0.252	0.002
CTX	0.228	0.002
Number of falls in the previous year	0.218	0.007
Charlson index	0.196	0.008
No. of medications	0.183	0.014
No. of comorbidities	0.173	0.019
Phosphorus	0.155	0.039
**Negative correlations**		
Stationary march	-0.344	0.000
eGFR	-0.335	0.001
MET	-0.255	0.002
Flamingo	-0.239	0.003
Albumin	-0.228	0.002

Spearman's test. Abbreviations: FGF23 – fibroblast growth factor 23; PTH – parathyroid hormone; P1NP – procollagen type 1 N-propeptide; CTX – C-terminal telopeptide of type 1 collagen; eGFR – estimated glomerular filtration rate; MET – energy expenditure.

**Figure 1:**
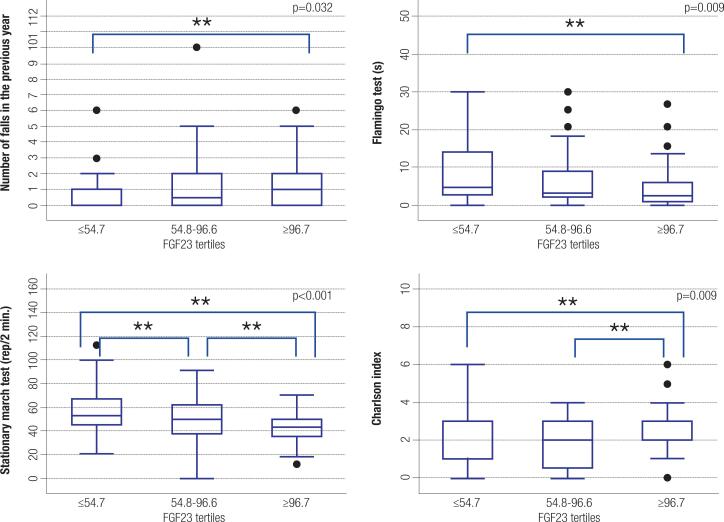
Relationship of FGF23 tertiles with the number of falls in the previous year, scores in the Flamingo and stationary march tests, and Charlson index (**p ≤ 0.05).

The individuals were divided into tertiles based on the FGF23 levels (≤54.77, 54.78-96.61, and ≥ 96.62 RU/mL). Those in the highest FGF23 tertile had had a greater number of falls in the previous year (p = 0.032), worse performance in the Flamingo (p = 0.009) and stationary march (p < 0.001) tests, and worse CI (p = 0.009) compared to those in the other tertiles (Figure 1).

**Figure 2:**
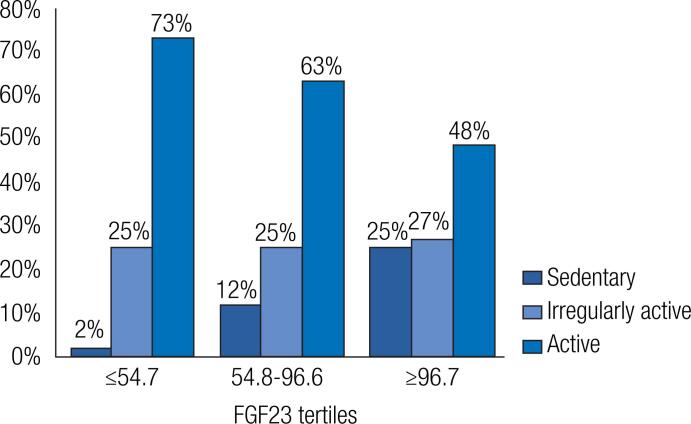
Distribution of very old individuals by International Physical Activity Questionnaire (IPAQ) categories according to FGF23 tertiles.

Figure 2 depicts the distribution of the participants by IPAQ categories according to tertiles of FGF23 concentrations. The highest tertile shows a trend towards a higher percentage of sedentary participants than the lowest tertile (p = 0.056).

Multiple regression models were tested to identify independent factors that could be associated with successful aging. Differences among FGF23 tertiles remained significant in the final models for the following markers: falls in the previous year (Supplementary Table 1), performance in the Flamingo and stationary march tests (Supplementary Tables 2 and 3, respectively), lean mass index, and IPAQ categories. Only eGFR remained significant in the final regression model for the CI.

In the final model for falls in the previous year (Supplementary Table 1), the following factors remained significant: age (individuals aged up to 85 years had on average 2.3 times fewer falls than those above 96 years), albumin (each 1.0 g/dL increase was associated with a 50% reduction in the number of falls), fat percentage (for each 10% increase in body fat, there was a 50% reduction in the number of falls), normal bone mass (individuals with normal BMD had 76% fewer falls than those with osteoporosis), and FGF23 (individuals in the lowest FGF23 tertile had 50% fewer falls than those in the highest tertile).

For the stationary march test (Supplementary Table 3), the factors that remained significant in the final model were male gender (men performed 10.2 more repetitions than women), age (for each 1-year reduction in age, there was an increase in one repetition), albumin (with each 1 g/dL increase in albumin level there was an increase of 15 repetitions), P1NP (for each 10 ng/mL decrease there was an increase of one repetition), absence of use of calcitriol (participants not taking calcitriol presented 18 more repetitions compared with those taking it), and FGF23 (individuals in the lowest FGF23 tertile presented 6.8 and 8.0 more repetitions than those in the middle and highest tertiles, respectively).

For the number of fractures in the previous year, only the BMI categories remained significant in the final model, and the participants with low weight presented 15 times more fractures than those with excess weight.

## DISCUSSION

The population evaluated in this study is singular, being all participants 80 years or older living independently in the community, and with chronic health conditions under control. As far we know, this is the first study to investigate FGF23 concentrations and their relationship with clinical data, physical performance, and falls in this population.

Serum concentrations of FGF23 in this population varied widely from 6.0 to 3,170.8 RU/mL (median 68.0 RU/mL), and in 22 of the individuals, the FGF23 levels were above 200 RU/mL (outliers). Although there were 3 individuals with primary hyperparathyroidism among them, other factors commonly associated with very high concentrations of FGF23 were not found in this subgroup of patients, who had eGFR above 32 mL/min, normal phosphorus levels, were not current smokers, and did not use calcitriol (19). That suggests that these high concentrations may be due to accumulated inactive C-terminal forms detected by the assay. Studies using the same type of assay on cohorts of younger and healthier individuals also found a nonparametric distribution of FGF23 concentrations, but with lower median values (between 13 RU/mL and 55.1 RU/mL) (20,21). The values obtained with assays that only detect the intact form of FGF23 are reported to have normal distributions (19,21). Regarding age, Weber and cols. found a significant positive correlation between FGF23 and age (20). In the same way, Souberbielle and cols. observed that individuals aged 60 years or more had higher mean FGF23 levels than individuals in other age groups. However, in their study the only parameter that remained significantlycorrelated with FGF23 after a multiple regression analysis was the 1,25(OH)_2_D, which was not measured in our cohort (19).

FGF23, along with its coreceptor klotho, is currently considered an important regulator of phosphorus homeostasis and of vitamin D metabolism (4). FGF23 is produced by osteocytes and osteoblasts and acts as a phosphaturic hormone in proximal tubules, while suppressing the synthesis and promoting the degradation of the active form of vitamin D (4). The earliest change found in chronic kidney disease (CKD) patients is a decrease in klotho expression, which subsequently leads to a state of resistance, with a compensatory increase in FGF23 preceding the increase in phosphatemia (3). In patients with CKD, high FGF23 concentrations have been associated with worse prognosis (22,23). In the general population, observational studies have also found an association between high levels of FGF23 and increased risk of cardiovascular disease, cardiovascular mortality, and noncardiovascular mortality, independently of the renal function (24,25).

In the present study, the relationships observed between higher levels of FGF23 and poorer neuromuscular performance were independent of the renal function, as observed in the number of falls in the previous year, in the Flamingo and stationary gait tests, and in the lean mass index. Although the expression of FGF23 has been identified in skeletal and cardiac muscle, the actions of FGF23 in the muscular system are still contradictory and little known (4,5). Some authors have found an association between FGF23 and striated muscle hypertrophy, both skeletal and cardiac (4,26), while other authors have suggested an inducing effect on cell senescence by an increase in the expression of tumor suppressor proteins P53 and P21 (27).

Studies have shown an inverse relationship between serum phosphate levels and muscle strength, linking hyperphosphatemia to a possible mechanism involved in sarcopenia (28,29). However, in our study, serum phosphorus levels did not remain statistically significant in any of the regression models that explained the relationships between the collected data and the results of performance in the physical tests, number of falls or fractures in the last year or even the lean mass. Thus, sarcopenia resulting from an inflammatory status linked to FGF23 becomes the more plausible explanation for this finding (4).

Recent cohort studies have shown an association between higher serum concentrations of FGF23 and frailty in elderly people, independently of phosphorus levels or other markers of mineral metabolism, both in patients predominantly without CKD (30) and patients with CKD (31). In the cohort of elderly patients with CKD, an association between higher serum concentrations of FGF23 and falls was observed (31), similarly to our study. In these large cohorts, however, the subjects were younger than ours [mean age 78 ± 4.7 years (30) and 73 ± 9 years (31)]. In the cohort of elderly patients without CKD (30), FGF23 measurements were performed with the same assay used in the present study and they found a median FGF23 value (70.3 RU/mL) similar to the one we found (68.0 RU/mL). In that cohort, the authors found association between higher concentrations of FGF23 and slow gait speed but not with grip strength (30). We hypothesized, just like these authors, that FGF23 may have a more prominent effect on proximal than distal muscle strength, perhaps via inhibition of active vitamin D [1,25(OH)_2_D]. Our study not only reinforces this hypothesis through the results of stationary gait (proximal muscle strength of lower limbs), but also shows FGF23’s correlation with static balance (Flamingo test). This finding corroborates the possibility that FGF23 exerts effects on sensory neurons (32,33).

In the present study, the reduced number of fractures in the previous year (n = 8, 5.2%) prevented further statistical analysis. However, when separated by FGF23 levels, fractures occurred in 15.8% (n = 3) individuals with FGF23 > 200 RU/mL but only in 3.8% (n = 5) of those with FGF23 < 200 RU/mL. Although this difference has not reached statistical significance, it may suggest some deleterious effect of high concentrations of FGF23 on the risk of fractures. The association between increased concentrations of FGF23 and fractures has already been described, both in populations of elderly men (34) and in populations with CKD (23). We found a positive association of FGF23 with PTH, CTX, and P1NP, which are markers known to be associated with high turnover and bone loss (35). Besides, most participants also had vitamin D deficiency, which added to a possible reduction in 1,25(OH)_2_D concentrations due to inhibition of 1-alpha-hydroxylase by FGF23. This could aggravate the hyperparathyroidism and its consequences on bone mineralization and contribute to the poor performance of individuals with elevated FGF23 concentrations.

Our study has several limitations, including its cross-sectional design, which prevented us from evaluating associations with future outcomes. With the absence of a control group, the conclusions of this study in very old individuals comprised of independent aging subjects cannot be extrapolated to other populations of younger or fragile elderly individuals. The assay used in the study measures the pool of circulating forms of FGF23, including inactive C-terminal forms, which may not adequately represent the normal physiological state. In addition, information on dietary calcium intake was not available, 1,25(OH)_2_D was not evaluated, and data on fractures were based on the patients’ history and were not necessarily documented. Despite these limitations, the findings of our study have great relevance in the current context, considering that life expectancy has grown exponentially in the world and data on this peculiar population of very old individuals are still scarce. Furthermore, as far as we know, these findings were the first to correlate FGF23 as an independent factor for balance and strength of lower limbs, properties that may predict falls, and separately evaluated.

In conclusion, our study demonstrated a relationship between increased levels of FGF23 and worse neuromuscular performance, demonstrated by the surrogate measures of falls during the previous year, scores in the Flamingo and stationary march tests, that especially evaluate muscle strength, aerobic capacity, and balance, independently of renal function. This may well be the first study measuring FGF23 concentrations in a community-dwelling, independent, very old individuals making correlations with these parameters. The results suggest that FGF23 could be a marker of physical performance in this population, indicating a potential role of FGF23 on musculoskeletal health.

## Appendix

**Supplementary Table 1:** Results of the initial and final Poisson model for the number of falls in the previous year

		Initial model		Final model	
		Average ratios (95% CI)	p	Average ratios (95% CI)	p
Age group (years) (ref.= ≤85)					
	86-90	0.85 (0.56; 1.29)	0.441	0.85 (0.58; 1.24)	0.395
	91-95	0.82 (0.49; 1.39)	0.465	0.80 (0.50; 1.30)	0.368
	96+	2.38 (1.31; 4.32)	0.005	2.29 (1.33; 3.93)	0.003
Male sex (ref. = Female)		1.41 (0.90; 2.20)	0.133	-	-
BMI (kg/m^2^)		1.002 (0.94; 1.067)	0.962	-	-
Albumin		0.52 (0.29; 0.9)	0.021	0.50 (0.29; 0.86)	0.013
eGFR		1.00 (0.98; 1.02)	0.900	-	-
% fat mass		0.97 (0.93; 1.01)	0.093	0.95 (0.93; 0.97)	<0.001
FGF23 (ref. = 1° Tertile)					
	2° tertile (54.8-96.6)	1.52 (0.97; 2.37)	0.066	1.48 (0.96; 2.28)	0.078
	3° tertile (≥96.7)	1.99 (1.23; 3.19)	0.005	2.03 (1.31; 3.15)	0.002
Diagnosis of osteoporosis (ref. = osteoporosis)					
	Normal	0.24 (0.09; 0.63)	0.004	0.26 (0.10; 0.65)	0.004
	Osteopenia	0.89 (0.61; 1.29)	0.542	0.96 (0.69; 1.34)	0.817
N		148		148	

Abbreviations: 95% CI – 95% confidence interval; BMI – body mass index.

**Supplementary Table 2:** Results of the initial and final linear regression model for Flamingo test

		Initial model		Final model	
		Coefficient (95% CI)	p	Coefficient (95% CI)	p
Age group (years) (ref. = ≤85)					
	86-90	-2.40 (-5.31; 0.50)	0.104	-2.55 (-5.44; 0.35)	0.085
	91-95	-6.68 (-10.68; -2.67)	0.001	-6.76 (-10.75; -2.77)	0.001
	96+	-6.74 (-14.05; 0.58)	0.071	-7.40 (-14.64; -0.16)	0.045
Albumin		2.50 (-1.97; 6.97)	0.271	-	-
P1NP		-0.02 (-0.08; 0.03)	0.455	-	-
FGF23 (ref. = 1° Tertile)					
	2° tertile (54.8-96.6)	-2.03 (-5.16; 1.1)	0.203	-2.16 (-5.28; 0.96)	0.174
	3° tertile (≥96.7)	-3.42 (-6.77; -0.07)	0.045	-4.06 (-7.28; -0.85)	0.014
N		151		151	
R^2^ (%)		15.32		14.09	
adjusted R^2^ (%)		11.17		11.13	

Abbreviations: 95% CI – 95% confidence interval; P1NP – procollagen type 1 N-propeptide; FGF23 – fibroblast growth factor 23. R2 – model determination coefficient.

**Supplementary Table 3:** Results of the initial and final linear regression model for stationary gait

		Initial model		Final model	
		Coefficient (95% CI)	p	Coefficient (95% CI)	p
Age		-1.09 (-1.71; -0.47)	0.001	-1.03 (-1.59; -0.47)	<0.001
Albumin		13.76 (4.34; 23.18)	0.005	15.09 (6.59; 23.6)	0.001
P1NP		-0.22 (-0.38; -0.05)	0.012	-0.11 (-0.22; -0.01)	0.037
% fat		-0.43 (-1.07; 0.20)	0.180	-	-
Number of comorbidities		-0.97 (-2.46; 0.52)	0.202	-	-
eGFR CKD (mL/min)		-0.07 (-0.28; 0.14)	0.525	-	-
BMD – Total Hip		5.72 (-18.34; 29.79)	0.639	-	-
Number of medications		-0.15 (-1.27; 0.97)	0.786	-	-
CTX		18.52 (-1.53; 38.57)	0.070	-	-
Male sex (ref. = Female)		6.26 (-2.03; 14.55)	0.138	10.15 (4.54; 15.76)	<0.001
Ethnicity (ref. = Caucasian)					
	African	0.20 (-11.43; 11.82)	0.973	-	-
	Asian	8.08 (-0.65; 16.82)	0.069	-	-
	Hybrid	0.96 (-6.28; 8.19)	0.794	-	-
FGF23 (ref. = 1° tertile)					
	2° tertile (54.8-96.6)	-5.74 (-12.2; 0.72)	0.081	-6.80 (-12.72; -0.89)	0.025
	3° tertile (≥96.7)	-6.21 (-13.61; 1.19)	0.099	-7.95 (-14.43; -1.48)	0.016
BMI		0.21 (-0.76; 1.17)	0.673	-	-
Use of Calcitriol (ref. = Not used)		-15.24 (-31.87; 1.38)	0.072	-17.90 (-33.46; -2.34)	0.024
N		145		148	
R^2^ (%)		40.40		34.84	
adjusted R^2^ (%)		32.43		31.58	

Abbreviations: 95% CI – 95% confidence interval; eGFR CKD – estimated glomerular filtration rate by the CKD-EPI formula; BMD – body mass density; CTX – C-terminal telopeptide of type 1 collagen; FGF23 – fibroblast growth factor 23; BMI – body mass index. R2 – model determination coefficient.
